# Asymmetries in lean mass, balance, and stability in 12-year-old female soccer (football) players: a cross-sectional study

**DOI:** 10.3389/fspor.2025.1705797

**Published:** 2025-11-27

**Authors:** Runa Stefansdottir, Hekla D. Gudmundsdottir, Erlingur Johannsson, Vaka Rognvaldsdottir

**Affiliations:** 1 Center of Sport and Health Sciences, School of Education, University of Iceland, Reykjavik, Iceland; 2 Department of Sport, Food and Natural Sciences, Western Norway University of Applied Sciences, Bergen, Norway

**Keywords:** youth, football, asymmetry, dual-energy x-ray absorptiometry (DXA), balance, female athletes, single-leg landing

## Abstract

**Background:**

Physical asymmetries in soccer (football) are inconsistently reported, and data on female youth players remain limited. This study evaluated asymmetries in leg lean mass, dynamic balance, landing kinetics and postural stability among 12-year-old female soccer players.

**Methods:**

Eighty-five players from 10 football clubs in Reykjavik, Iceland, participated between April and June 2024. Leg lean mass was assessed via dual-energy x-ray absorptiometry (DXA), dynamic balance using the Y Balance Test (YBT), and single-leg landing test with VALD force plates. Asymmetry between the preferred kicking leg (PKL) and the non-preferred kicking leg (NPKL) was assessed with paired t-tests. Absolute asymmetry magnitudes were compared across coach-rated performance groups using Welch's ANOVA.

**Results:**

Participants had a mean age of 11.7 ± 0.3 years, height of 154.4 ± 6.9 cm, and weight of 44.2 ± 8.1 kg. No significant asymmetries were found between leg lean mass (*p* = 0.197), landing force (*p* = 0.905) or time to stabilization (*p* = 0.083). However, significant asymmetry was observed in anterior reach on the YBT (*p* = 0.008, *d* = 0.29), favoring the NPKL. No differences were seen in other YBT directions.

**Discussion:**

While lean mass and landing performance were symmetrical, the anterior reach asymmetry may reflect early neuromuscular differences. These results suggest that asymmetries in young female players may begin to emerge in select functional domains, even in the absence of structural or kinetic differences. Ongoing monitoring during adolescence may help clarify how training exposure and development influence these patterns.

## 1 Introduction

Soccer (football) is the most popular sport worldwide, with female participation growing rapidly over the past two decades. In 2023, global female participation in soccer was estimated at 30 million players, and this number is expected to continue rising (1–3). Iceland has a strong soccer culture, with the women's national team ranked 14th in the FIFA Women's World Rankings as of June 2025 (4). In youth soccer, unlike many countries, Iceland does not use exclusive academies or early talent selection. Instead, players of all ability levels train together in mixed groups within local clubs throughout development. This community-based model may support broad participation for all levels of players during early adolescence.

Despite the rise in female participation, research on youth female soccer players, particularly during early adolescence, remains limited (5). Puberty has a significant impact on physical development in youth athletes, with girls typically entering this stage earlier than boys (6). This earlier onset can lead to rapid increases in muscle mass, strength, and body size, which may affect training adaptation and performance potential (7, 8). While these developmental changes have been studied extensively among young male athletes, young female athletes are significantly underrepresented in the literature (9–11). This disparity is likely rooted in historically lower female participation in sports and limited research attention to female developmental stage in sports (12).

Most soccer players exhibit a clear preference for one leg when kicking the ball, typically referred to as the preferred kicking leg (PKL), meanwhile the non-preferred leg (NPKL) is therefore, more used for support and stability (13–15). Over time, this functional specialization between PKL and NPKL can lead to measurable asymmetries in strength, coordination, and loading patterns, particularly in sports that emphasize unilateral movement (16, 17). In adolescent and adult male soccer players, such asymmetries have been associated with performance impairments (e.g., reduced sprint speed, jump height) and increased injury risk, particularly when the asymmetry magnitude exceeds 10%–15%, a threshold commonly cited in the literature (18–21). Similar patterns have also been reported in adult female players, though they have been studied less frequently (22). One study by Bishop et al. found that interlimb asymmetries were associated with reduced sprint and jump performance in elite youth female players (18). However, recent systematic reviews have highlighted that the relationship between asymmetries and injury risk is not always consistent, with some studies reporting no clear link (23, 24). Given these mixed findings and the lack of data on younger, non-elite female soccer players, further work is needed to understand when and how such asymmetries begin to emerge, and whether they represent functional adaptations.

Performance-based assessments such as the Y Balance Test (YBT) and single-leg landing tests offer insight into neuromuscular control and postural stability (25, 26), but they do not capture potential structural asymmetries, such as differences in limb-specific muscle mass. Dual-energy x-ray absorptiometry (DXA) offers a validated, non-invasive method for quantifying these structural differences, yet its use in youth female athletes remains rare (27).

In early adolescence, monitoring side-to-side variations in muscle mass and function may provide insight into physical development and training adaptation. The present study aimed to assess asymmetries in lean mass, dynamic balance, landing force, and postural stability between the PKL and NPKL in 12-year-old female soccer players. Given that training exposure may vary across players within community-based teams, we also explored whether asymmetry magnitude differed by coach-rated performance rank.

## 2 Methods

### 2.1 Study design and participants

**Figure 1:**
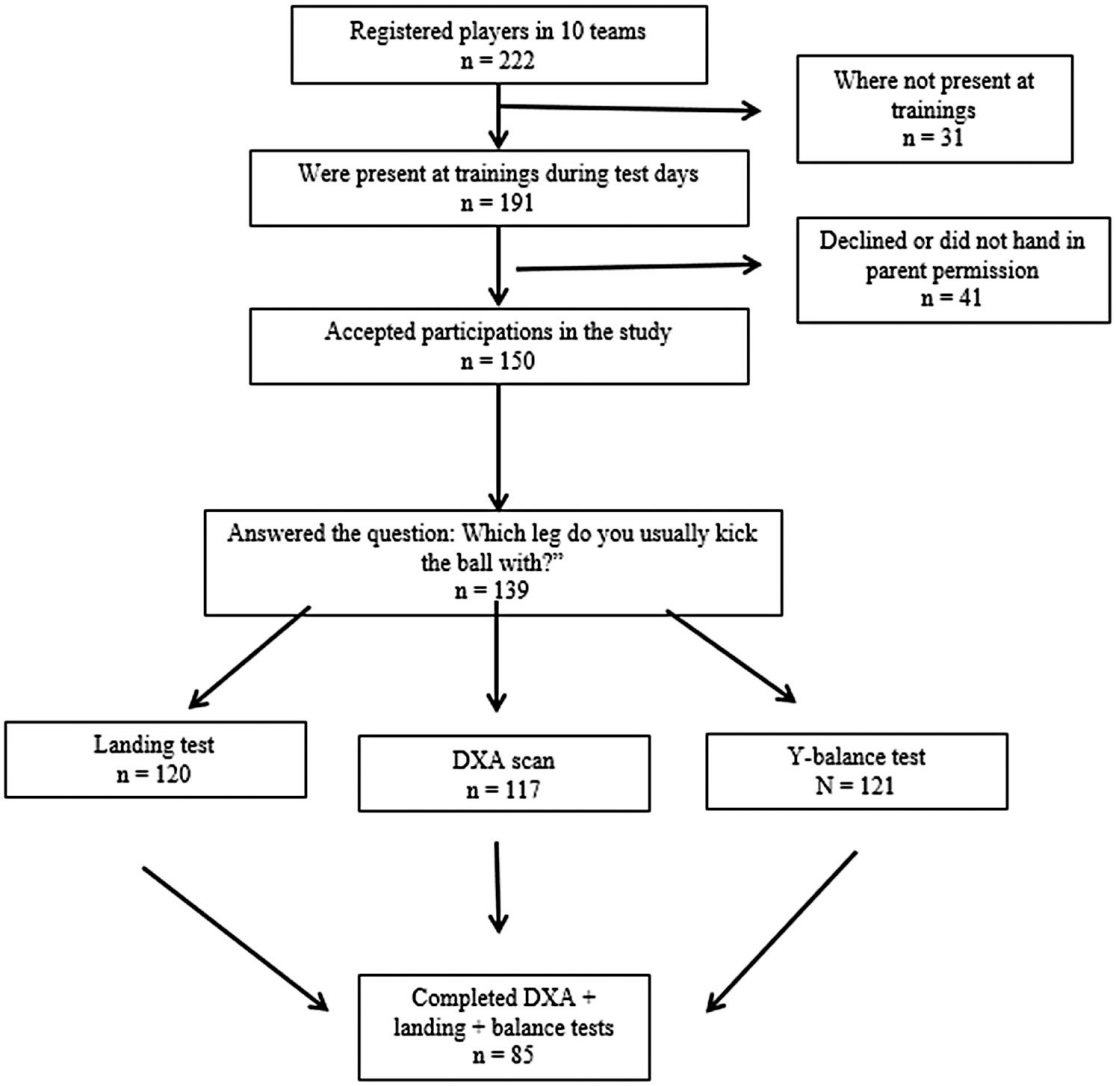
Flowchart of study participation.

This cross-sectional study utilized baseline data from the SKORA (Soccer Knowledge for Optimal Resilience and Athletic Performance in Female Youth) project, a longitudinal initiative examining the development of young female soccer players in Iceland, to investigate neuromuscular asymmetries in lean mass, balance, and landing mechanics at the initial data collection point. The participants were girls born in 2012 who all trained with football clubs in the Reykjavík metropolitan area. Of the 12 invited teams, selected to ensure geographic and competitive diversity, 10 agreed to participate. At the time of recruitment, 222 players were registered across the 10 clubs, of whom 150 chose to participate, giving a participation rate of 78.5%. Of these players, 85 had valid data for DXA scanning, single-leg landing tests, and the YBT. The participation flowchart is presented in Figure 1.

### 2.2 Data collection

The data collection was approved by the National Bioethics Committee and the Icelandic Data Protection Authority (Ref: VSN-24-023) and took place during the spring of 2024. Each participating club was assigned two data collection days, with larger teams allocated additional sessions when needed. Testing took place during regular team practices to minimize disruption to the players' daily routine. The first data collection day included questionnaire administration and single-leg landing tests, while the second day focused on dynamic balance using YBT. The testing order was randomized based on athlete availability during practice. All assessments were conducted indoors on level, hard flooring. The participants completed a standardized warm-up supervised by the research team before each test. DXA scans were conducted between 1 and 8 weeks after the field-based performance tests, specifically between May 23 and June 7, 2024. This delay was due to the late arrival and setup of the DXA scanner, which did not become operational until late May. Participants were scheduled for scans as soon as the equipment was available.

### 2.3 Questionnaire and performance level

The participants completed a standardized questionnaire designed for their age group. This study used the question “Which leg do you usually kick the ball with?” (response options: “Right”, “Left,” or “Both”). This question was used to determine each participant's PKL and NPKL. In the valid dataset of 85 subjects (Figure 1), 80 reported using their right leg and 5 their left leg as the preferred kicking limb. Consequently, the findings primarily reflect asymmetrical patterns in right-footed players.

### 2.4 Coaches' performance rank

Coaches were asked to rank each player based on performance using a 3-point scale:

Rank 1: Usually plays with the first teams (best team) or has the ability to do so.
Rank 2: Usually plays with the second team or has the ability to do so.
Rank 3: Usually plays with the third team or has not yet been assigned to a team.

All rankings were made independently by the participants' own team coaches, who were familiar with the players' performance across the season. While formal inter-rater reliability was not assessed, the rank categories were clearly defined in advance, and no players were ranked by multiple coaches. As such, intra-club consistency was maintained, and the rank variable reflects each coach's evaluation within their specific team context.

### 2.5 Physical performance tests

#### 2.5.1 Landing test

Landing force and postural stability were assessed with a single-leg drop landing test, performed on force plates (Vald Performance, Brisbane, Queensland, Australia). The force plates have been evaluated for accuracy for both jump and landing tests (28). Before testing, the participant stood still on the plates to calibrate baseline body weight. Each test began from a 30 cm–high platform. The participant was instructed to stand with their hands on their hips, step forward off the box, and land on one leg in the center of the force plate and maintain their balance for a minimum of 2–3 s or until given a cue to step off the plate. Each leg was tested three times in an alternating order, regardless of leg dominance, and a rest interval of approximately 30 s was provided between trials to minimize fatigue. The variables recorded included time to stabilization (seconds) and peak drop landing force (Newtons). The average of three tests per leg was calculated using the Vald Hub software. Interlimb asymmetry magnitude was calculated as the absolute difference between the values of the PKL and NPKL.

#### 2.5.2 Y balance test

Dynamic balance was assessed using the lower quarter *Y* balance test (Move2Perform, Evansville, IN, USA) with standardized equipment from Functional Movement Systems. Before testing, leg length was measured from the anterior superior iliac spine to the medial malleolus with the participant lying supine. During testing, the participant stood on a central footplate and used the opposite leg to push a block in three directions: anterior (AT), posteromedial (PM), and posterolateral (PL). Each direction was tested three times per leg before changing the standing leg and the maximal reach for each direction was used for analysis (29). Reach distances, were recorded in centimeters and normalized to leg length, and were represented as percentages (30).

$$\begin{array}{c} \mathrm{AT}\text{\%}=(\mathrm{AT}\;\mathrm{distance}/\mathrm{leg}\;\mathrm{length})\times 100\\ \mathrm{PM}\text{\%}=(\mathrm{PM}\;\mathrm{distance}/\mathrm{leg}\;\mathrm{length})\times 100\\ \mathrm{PL}\text{\%}=(\mathrm{PL}\;\mathrm{distance}/\mathrm{leg}\;\mathrm{length})\times 100 \end{array}$$

A composite score (CS) was calculated as a percentage of all directions of the YBT as follows (30, 55):

CS%=([ATdistance+PMdistance+PLdistance]/[3×leglength])×100

Asymmetry magnitude was defined based on the absolute percentage difference between the PKL and NPKL for the AT, PM, and PL directions, as well as the CS. The YBT has effectively been used in younger and older populations as a screening tool (29, 30).

#### 2.5.3 DXA scan

Body composition was assessed using a Lunar iDXA Pro Advance scanner (GE Healthcare, Chicago, IL, USA) at The Icelandic Heart Association (Hjartavernd). All DXA scans were conducted by certified radiology technicians, with all DXA appointments scheduled in the afternoon to ensure consistency. The use of DXA scan in estimating skeletal muscle mass in research settings for sport medicine has been validated for accuracy and reliability for young people (31, 32). The participant lay supine while the scanner passed overhead, capturing whole-body images for approximately 15–20 min. From DXA data, following body composition metrics were calculated; body fat percentage (Body fat%), fat free mass (FFM), trunk lean mass and leg lean mass. Regional lean mass values were extracted by side based on PKL and NPKL to evaluate magnitude of asymmetry in lean mass between sides.

### 2.6 Statistical analysis

All data were analyzed using Jamovi (version 2.3.28), and a *p* < 0.05 was considered statistically significant. The participants' characteristics and performance outcomes are descriptively reported as means ± standard deviations (SDs) and ranges. Data were screened for distributional assumptions prior to inferential testing. Normality was evaluated using the Shapiro–Wilk test and visual inspection of Q–Q plots and histograms. Performance variables were compared between the PKL and NPKL using paired samples *t*-tests, including lean mass, YBT score, landing force, and time to stabilization. The normality assumption was evaluated for two variables showing deviations from normality in the Shapiro–Wilk test. Given the large sample size (*N* = 85) and the known robustness of the paired *t*-test to moderate non-normality, parametric analyses were retained. To verify robustness, Wilcoxon signed-rank tests were additionally performed. The non-parametric results yielded the same significance pattern as the *t*-tests; therefore, only the *t*-test results are reported, with the Wilcoxon outcomes noted where relevant.

Asymmetry magnitudes were computed as the absolute difference between sides (PKL and NPKL). Whether these interlimb differences varied by coach-rated performance rank was examined using Welch's ANOVA. Descriptive analysis indicated that variables indicating limb differences were generally right skewed, as expected from the use of absolute values. Variables with strong right skewness, were transformed (square root) to improve normality and homogeneity of variances prior to Welch's ANOVA. The Welch ANOVA results based on transformed data did not differ from those obtained with the original values (both analyses yielded non-significant results). Therefore, to facilitate interpretation, results are presented for the untransformed variables.

## 3 Results

The participants' ages and body compositions are presented descriptively in Table 1. Their mean age was 11.7 ± 0.3 years, mean height was 154.4 ± 6.9 cm, mean weight was 44.2 ± 8.1 kg, and mean fat-free mass was 32.2 ± 4.8 kg.

**Table 1:** Participants’ ages and body compositions.

Characteristics	Mean	SD	Range (min–max)
Age (years)	11.7	0.322	11.3–12.2
Height (cm)	154.4	6.929	136.0–169.8
Weight (kg)	44.2	8.053	28.8–72.0
BMI (kg/m^2^)	18.4	2.57	14.9–27.1
FFM (kg)	32.2	4.804	21.1–44.4
Body fat (%)	26.5	5.937	14.5–45.2
Trunk lean mass (g)	14,449.1	2,190.7	9,296.9–19,993.5
Leg lean mass (g)	10,340.5	1,797.7	6,385.8–14,803.8

SD, standard deviation; BMI, body mass index; FFM, fat-free mass.

Comparisons of lean mass, YBT results, and landing test results between the sides of PKL and NPKL are presented in Table 2. The whole body lean mass did not differ significantly between the PKL (15,214.2 ± 2,250.3 g) and NPKL (15,269.1 ± 2,364.7 g) sides of the body (*p* = 0.448). Similarly, leg lean mass did not differ significantly between the PKL (5,185.1 ± 903.6 g) and NPKL (5,154.8 ± 907.5 g) sides (*p* = 0.197).

**Table 2:** Comparison of lean mass, balance test results, and landing test results between the PKL and NPKL sides.

Variables	Mean	SD	Range (min–max)	*p*-value
Muscle mass				
Lean mass: PKL (g)	15,214.2	2,250.3	9,983.5–21,575.2	0.448
Lean mass: NPKL (g)	15,269.1	2,364.7	9,958.0–20,720.6	
Leg lean mass: PKL (g)	5,185.7	903.6	3,316.5–7,618.5	0.197
Leg lean mass: NPKL (g)	5,154.8	907.5	3,069.3–7,455.2	
Y balance test				
AT: PKL (%)	59.80	4.76	44.0–72.0	
AT: NPKL (%)	61.09	5.57	48.0–74.5	**0**.**008**
PM: PKL (%)	93.56	7.32	75.5–110.5	
PM: NPKL (%)	94.39	7.4	72.0–112.0	0.229
PL: PKL (%)	91.81	6.96	69.0–107.5	
PL: NPKL (%)	91.49	6.74	71.5–109.0	0.619
CS: PKL (%)	98.25	7.24	72.22–115.98	
CS: NPKL (%)	98.91	7.37	75.92–119.02	0.155
Landing test				
Peak landing force: PKL (*N*)	1,496.53	487.09	528–612	
Peak landing force: NPKL (*N*)	1,494.41	530.06	485–3,152	0.945
Time to stabilization: PKL (s)	0.46	0.17	0.25–1.05	
Time to stabilization: NPKL (s)	0.50	0.21	0.21–1.41	0.083

SD, standard deviation; AT, anterior; PM, posteromedial; PL, posterolateral; CS, composite score; N, Newtons.

Bold values indicate statistical significance (*p* < .05).

In the YBT, the reach differed significantly between the limbs in the AT direction (*p* = 0.008, *d* = 0.29), with the NPKL reaching farther than the PKL (61.09% ± 5.57% vs. 59.8% ± 4.76%), but not in the PM (*p* = 0.229) or PL (*p* = 0.619) directions. In the landing test, peak landing force (*p* = 0.905) and time to stabilization (*p* = 0.083) did not differ significantly between limbs. The magnitude of asymmetry (calculated as the absolute difference between limbs) across player performance ranks is presented in Table 3. Whole-body lean mass asymmetry differed significantly between groups, with players ranked 1 exhibiting greater asymmetry magnitude than those ranked 2 and 3 (*p* = 0.005, *η*^2^ = 0.083). Leg lean mass asymmetry magnitude did not differ significantly between ranks (*p* = 0.414). In the YBT, the greatest asymmetry magnitude was seen in the AT direction among players ranked 1, although the difference between ranks was not significant (*p* = 0.087). Asymmetry magnitude in the PM and PL directions, as well as in the CS, did not differ significantly across ranks. In the landing test, asymmetry magnitudes for peak force and time to stabilization also did not differ significantly across ranks.

**Table 3:** Differences between the PKL and NPKL sides by coaches’ performance rank.

Variables	*N*	Rank	Mean	SD	*p*-value
*Δ* Lean mass (g)	40	1	597.73	461.36	**0**.**005**
	27	2	582.44	309.56	
	18	3	314.32	270.13	
*Δ* Leg lean mass (g)	40	1	156.84	102.88	0.414
	27	2	208.08	181.16	
	18	3	158.02	124.26	
*Δ* AT (%)	40	1	4.06	2.91	0.087
	27	2	3.78	2.8	
	18	3	2.55	2.16	
*Δ* PM (%)	40	1	4.54	3.6	0.448
	27	2	5.58	3.76	
	18	3	5.72	4.49	
*Δ* PL (%)	40	1	4.70	3.51	0.873
	27	2	4.56	4.23	
	18	3	4.22	3.08	
*Δ* CS (%)	40	1	3.36	2.07	0.511
	27	2	3.99	2.5	
	18	3	3.23	2.76	
*Δ* Time to stabilization (s)	40	1	0.14	0.13	0.154
	27	2	0.22	0.23	
	18	3	0.11	0.13	
*Δ* Peak landing force (*N*)	40	1	208.45	189.02	0.968
	27	2	202.33	157.29	
	18	3	192.05	248.13	

*Δ*, absolute difference between PKL and NPKL sides; *N*, number; SD, standard deviation; *N*, Newtons; CS, composite score from the YBT.

Bold values indicate statistical significance (*p* < .05).

## 4 Discussion

This study explored interlimb asymmetries in lean mass, dynamic balance, and landing performance among 12-year-old female soccer players in Iceland. The findings revealed largely symmetrical development between limbs, with no significant differences in lean mass, peak landing force, or time to stabilization between the PKL and NPKL. However, significant asymmetry was observed in the AT direction in the YBT, where the NPKL reached farther.

Limb preference is common in soccer, with the PKL typically used for ball striking and the NPKL for support and stabilization (33, 34). Given soccer's unilateral movement demands, it is often assumed that interlimb asymmetries develop over time (35, 36). However, in this study, no significant asymmetries in lean mass or landing performance were found, likely reflecting the participants' young age and relatively uniform training exposure. These findings are consistent with those by Raya-González et al., who reported minimal asymmetries and no functional impact in slightly older female players (37). Pubertal hormonal changes, particularly increases in growth hormone and estrogen, promote muscle hypertrophy and lean mass gains, supporting neuromuscular development in adolescent athletes (7, 8). These processes typically explain the increases in lean mass observed during growth and maturation. However, given the age of our sample, most participants were likely pre-adolescents, and such adaptations may not yet be present. While some research suggests the NPKL may be stronger or more stable due to its support role (14, 38), this is primarily observed in older male athletes and may not apply here. Our findings provide an early baseline for muscular development and symmetry in female youth players and support the study's aim of evaluating asymmetries at a key developmental stage. While all participants were of similar chronological age, individual variation in biological maturation likely existed. Pubertal development, including growth spurts and menarche onset, could influence lean mass, balance, and neuromuscular coordination (7, 8).

The only significant asymmetry identified was in the AT direction of the YBT, where the NPKL reached farther than the PKL. No significant differences were observed in the PM or PL directions. This directional pattern is consistent with previous studies in similarly aged athletes (36, 39), suggesting that AT asymmetry may emerge earlier, while differences in PM and PL may develop later with maturation and training. This likely reflects ongoing development of postural control and neuromuscular coordination, which improve with age and sport-specific exposure (40, 41). At 12 years old, training loads and specialization levels are typically low, which may limit the emergence of widespread asymmetries. Moreover, the magnitude of this asymmetry was relatively small (mean difference ∼1.3%) and below commonly cited clinical thresholds for concern (e.g., >4 cm or ∼4% in YBT anterior reach) (30). Therefore, this asymmetry is unlikely to reflect a functional deficit or injury risk marker at this stage. Instead, it may represent normal developmental variability. However, the anterior reach direction is often the first to show asymmetry in youth athletes, and ongoing monitoring may help identify individuals with emerging functional imbalances. From a practical standpoint, this finding supports incorporating early balance training that addresses both limbs equally to promote symmetrical neuromuscular control. This is particularly important given the potential long-term effects of early specialization, including increased injury risk and sport dropout (42–45).

The average CS on the YBT in this sample exceeded the 89.6% threshold previously associated with elevated injury risk in older athletes (46). However, around 10% of players on both the PKL and NPKL sides fell below this clinical cutoff, suggesting that a small subset may have reduced dynamic balance. These individuals could potentially benefit from early neuromuscular training to improve single-limb stability. Balance and unilateral control are often underemphasized in preadolescent training (47, 48), yet this stage may offer a valuable window for targeted intervention. That said, more recent research has raised questions about the reliability of fixed asymmetry or CS thresholds for injury prediction. For example, a systematic review by Helme et al., found inconsistent associations between asymmetry and injury risk across soccer-related studies: only half showed clear links, while others reported no or partial associations (23). Additionally, most of these studies were conducted in male populations, limiting relevance for youth female players. Overall, while functional asymmetry may be one risk factor, it should be interpreted in the context of other variables, such as neuromuscular control, movement quality, and training load, rather than as a standalone predictor of injury.

Landing performance did not differ significantly between limbs, suggesting that neuromuscular control and postural stability are still developing symmetrically at this age. The single-leg drop landing test from a 30 cm platform is a validated screening tool for dynamic stability deficits, especially those linked to noncontact injuries like ankle sprains (25). However, most research using this test has focused on older or previously injured athletes (49), while the present sample was uninjured and relatively early in their training trajectory. These factors may explain the lack of detectable asymmetries. Our findings are partially consistent with Wang and Fu, who also reported symmetrical landing kinetics in female collegiate players but observed lower postural stability in the nondominant leg (50). In contrast, no such asymmetry was found here, likely reflecting the limited specialization and balanced training exposure in 12-year-olds. At this age, athletes typically engage in generalized movement preparation rather than targeted unilateral training (51), which may help prevent early asymmetry. As sport-specific loads increase in adolescence, continued monitoring of postural control and neuromuscular coordination will be important to identify emerging imbalances.

Leg lean mass was symmetrical between the PKL and NPKL, consistent with other physical measures, despite the unilateral nature of soccer. This supports the idea that structural adaptations, such as side-specific muscle development, may not emerge until later in adolescence, when cumulative training loads become higher (52). Because few studies have examined females at this stage of development, it remains uncertain how and when such asymmetries arise.

A small but significant difference was observed in whole-body lean mass asymmetry across coach-rated performance ranks: players ranked 1 showed greater asymmetry magnitude than those ranked 2 or 3. However, this difference was not present in leg lean mass or any functional tests (balance or landing), suggesting limited functional relevance. One possible explanation is that higher-ranked players are exposed to greater training loads and match play, which could gradually contribute to slight structural imbalances. Nonetheless, these results must be interpreted cautiously, as coach-assigned ranking combines observed skill and perceived potential, and thus remains a subjective measure rather than an objective indicator of performance (53). In the Icelandic youth soccer context, where players of all ability levels train together, such categorizations should be viewed as approximate rather than definitive indicators of talent. In practical terms, this observation suggests that lean mass asymmetry magnitude in itself may have limited utility for identifying talent, especially compared to performance-based measures such as high-intensity endurance capacity (54).

### 4.1 Strengths and limitations

A key strength of the study is its use of validated, objective measurements, including DXA scans, force plates, and standardized balance testing. The sample size was also relatively large for this age group, and it represents one of the first studies to explore asymmetry in young Icelandic female soccer players. However, it also had some limitations. Firstly, its cross-sectional design limited our ability to infer causality or track developmental trends over time. Further, the sample was highly unbalanced according to preference of kicking leg, as 80 participants considered their right foot as PKL with only five considered their left foot as their PKL, which limits the statistical power to detect effects related to footedness and restricts the generalizability of the findings. Consequently, the present results should be interpreted as primarily representative of asymmetry patterns among right footed, observed via PKL, athletes. Future studies would benefit from including a more balanced distribution of right- and left-footed participants and employing multidimensional measures of limb dominance, combining self-report with task specific or performance-based assessments. Some participants had difficulty performing the more complex motor tasks, particularly the landing and balance tests, which may have affected test scores. Despite standardized verbal instructions and demonstrations, varying levels of comprehension and execution were observed among the participants, requiring individualized feedback and potentially introducing performance variability. Testing took place in indoor environments where waiting times may have introduced fatigue or nervousness, especially given the young age of our participants. Lastly, a 1–8 week gap occurred between field testing and DXA scanning due to the late arrival and setup of the DXA scanner. Although no formal strength training took place during this period, soccer training inherently includes sprinting, accelerations, decelerations, and directional changes. As a result, minor fluctuations in body composition cannot be entirely ruled out, and we acknowledge this time lag as a limitation of the study.

## 5 Conclusions

While most physical measures were symmetrical at this stage of development, subtle functional asymmetries, particularly in anterior reach, may reflect early neuromuscular differences. Monitoring these patterns through adolescence could inform age-appropriate training strategies that support movement efficiency and healthy development as training loads increase. Given the limited research focusing on young female soccer players, our study offers valuable insights into physical development and functional symmetry in this population, enabling coaches and practitioners to tailor training approaches accordingly.

## Data Availability

The raw data supporting the conclusions of this article will be made available by the authors, without undue reservation.
